# Cancer-derived immunoglobulin G promotes LPS-induced proinflammatory cytokine production via binding to TLR4 in cervical cancer cells

**DOI:** 10.18632/oncotarget.2359

**Published:** 2014-08-23

**Authors:** Juping Wang, Danyi Lin, Hui Peng, Jimin Shao, Jiang Gu

**Affiliations:** ^1^ Department of Pathology and Pathophysiology, Zhejiang University School of Medicine, Hangzhou China.; ^2^ Provincial Key Laboratory of Infectious Diseases and Molecular Immunopathology, Department of Pathology, Shantou University Medical College, Shantou, China.

**Keywords:** IgG, cancer, promote, TLR4, proinflammatory cytokine

## Abstract

Numerous studies have shown that various cancer cells express immunoglobulin G (IgG). However, the function of cancer-derived IgG and the underlying mechanism remain unclear. In this study, we demonstrated that IgG expression was significantly altered after exposure to LPS in cervical cancer cells, suggesting that IgG was potentially involved in regulation of TLR4 signaling. Reduction of IgG attenuated LPS-induced proinflammatory cytokine production. The phosphorylation levels of NF-κB and MAPK were consistently suppressed by knockdown of IgG, which in turn impaired NF-κB nuclear translocation and the activity of NF-κB responsive element. Furthermore, we found that IgG was recruited to TLR4 in the cytoplasm after LPS stimulation, and IgG silencing inhibited LPS-initiated proinflammatory cytokine production through downregulating TLR4 expression. Similar results were obtained in a mouse model of endotoxemia and human tissues. Taken together, our findings demonstrate that IgG is a positive regulator of LPS-induced proinflammatory cytokine production by binding to TLR4 and enhancing its expression. TLR4 signaling plays a positive role in the development of many inflammation induced cancers such as cervical cancer. Our study strongly indicates that IgG may promote cervical cancer cell proliferation through enhancing TLR4 signaling. IgG may be a novel therapeutic target in treating inflammation mediated cancers.

## INTRODUCTION

Toll-like receptors (TLRs) play a key role in innate immune system by recognizing conserved components of invading microbial pathogens [1-3]. So far, 13 distinct TLRs have been found in murine and human. In addition, members of the TLR family have been discovered in evolutionarily distant organisms such as fish and plants [4-6]. Among TLRs, TLR4, a critical member, initiates innate immune responses by activating signaling pathways dependent on the adaptor protein myeloid differentiating factor 88 (MyD88) or the adaptor toll/il-1r domain-containing adaptor inducing interferon-β (TRIF). The binding between TLR4 and MyD88 finally leads to the activation of nuclear factor κB (NF-κB) and mitogen-activated protein kinase (MAPK) pathways and induces the production of proinflammatory cytokines such as tumor necrosis factor-α (TNF-α), interleukin-6 (IL-6), and IL-1β. While the interaction between TLR4 and TRIF finally activates interferon regulatory factor 3 (IRF3) and produces type I interferon (IFN) such as IFN-α and IFN-β [7, 8]. Recent studies showed that TLR4 was expressed not only on immune cells but also on cancer cells such as cervical cancer [9-11], lung cancer [12, 13], and breast cancer [9]. TLR4 expression on various cancer cells suggests that TLR4 signaling may play a crucial role in cancer development and immunity.

Only B lymphocytes and plasma cells are capable of producing IgG in classical theory. However, increasing evidences show that various types of cancer cells such as cervical cancer, colon cancer, and breast cancer express and secrete IgG [14-16]. Compared with B lymphocyte-produced IgG, cancer-derived IgG has the following unique characteristics: (1) Cancer-derived IgG gene exhibits specific restricted patterns of the heavy chain variable segment (V_H_), diversity segment (D), and joining segment (J_H_) recombination (V_H_DJ_H_), which are distinct from the unlimited diversity of B lymphocyte-produced IgG gene [17]. (2) The regulatory mechanism for IgG gene expression in cancer cells is different from that in B lymphocytes. IgG promoter is activated in cancer cells. Further more, two novel positive regulatory elements, one at -800 to -610 bp and another at -610 to -300 bp, are confirmed in cancer cells. The transcriptional factor, which plays a crucial role in IgG gene transcription in cancer cells, is not octamer-related protein-2 (Oct-2) but Oct-1[18]. (3) Glycosylation patterns of cancer-derived IgG are quite different from those of B lymphocyte-produced IgG. B lymphocyte-produced IgG has no O-linked glycans, and has only one N-glycosylation at N297 position of IgG heavy chains and terminal N-acetylneuraminic acid (NeuAc), while cancer-derived IgG has not only O-linked and N-linked glycans but also terminal NeuAc and N-glycolylneuraminic acid [19]. (4) Cancer-derived IgG shows much lower immunoactivity, compared to B lymphocyte-produced IgG [20]. Nevertheless, the functions of cancer-derived IgG have rarely been reported. Our previous studies confirmed that cancer-derived IgG can enhance growth and proliferation of cancer cells via inducing production of reactive oxygen species at low level *in vitro* and *in vivo* [21]. Niu et al reported that blockage of IgG increased apoptosis and invasion of colorectal cancer [15]. However, it is not known whether or how IgG interacts with TLR4 signaling in cancer cells. In this study, we first confirmed that IgG interacted with TLR4 after lipopolysaccharide (LPS) stimulation and enhanced its expression in cervical cancer cells that significantly strengthened LPS-induced activities of NF-κB and MAPK including extracellular signal-regulated kinase (ERK), c-Jun N-terminal kinase (JNK), and p38, which in turn increased the production of proinflammatory cytokines including TNF-α, IL-6, and IL-1β. In brief, our study demonstrates a new function of cancer-derived IgG as a positive regulator of LPS-induced proinflammatory cytokine production, thus providing new insight into the fine tuning of TLR-triggered innate inflammatory responses in cervical cancer cells.

## RESULTS

### Kinetics of IgG expression in LPS-stimulated cervical cancer cells

**Figure 1:**
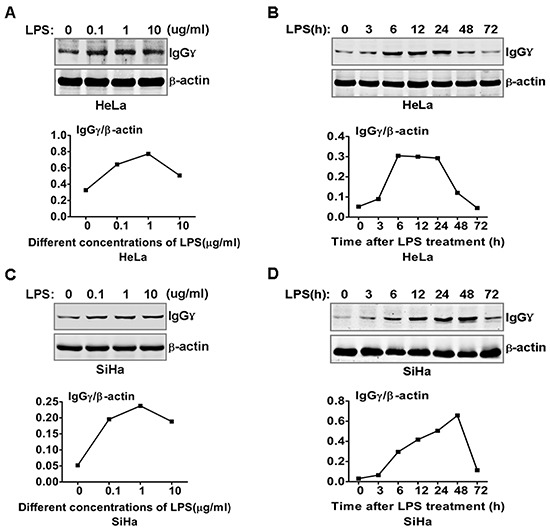
LPS regulated IgG expression in cervical cancer cells HeLa cells **(A)** and SiHa cells **(C)** were stimulated with the indicated doses of LPS for 24 h. IgGγ expression was detected with immunoblot. HeLa cells **(B)** and SiHa cells **(D)** were stimulated with 100 ng/ml LPS as indicated. IgG expression was detected with immunoblot. The shown results are representative of three independent experiments. The quantified results of IgGγ expression were shown in the lower panel. The values were normalized to the β-actin signal.

To explore the role of IgG in cervical cancer cells response to LPS stimulation, we examined whether IgG expression could be induced by LPS in cervical cancers cells. The results showed that different doses of LPS increased significantly IgGγ expression in HeLa cells (Fig. 1A). However, a higher dose of LPS (10 μg/ml) did not increase further the expression level of IgGγ. To investigate the kinetics of the regulation of IgGγ expression in HeLa cells induced by LPS, we examined IgGγ expression in HeLa cells treated with LPS for different time periods with immunoblot and real-time quantitative reverse transcription PCR (RT-qPCR). The results indicated that IgGγ expression reached the peak level after LPS treatment for 6 h, then gradually decreased to the normal level (Fig. 1B). The kinetical change of IgGγ expression in mRNA level was different from that of protein level in HeLa cells (data not shown). The same experiments were performed in SiHa cells. The results showed that the kinetics of IgGγ expression after treatment with different doses of LPS in SiHa cells was similar to that in HeLa cells (Fig. 1C). For the kinetics of IgGγ after LPS treatment for different time periods, IgGγ expression reached the peak level after LPS treatment for 48 h in SiHa cells, which was different from that of HeLa cells (Fig. 1D). The above kinetical change of IgGγ expression in mRNA level was different from that of protein level in SiHa cells (data not shown).

### Silencing of IgG inhibits the production of LPS-induced proinflammatory cytokines in cervical cancer cells

**Figure 2:**
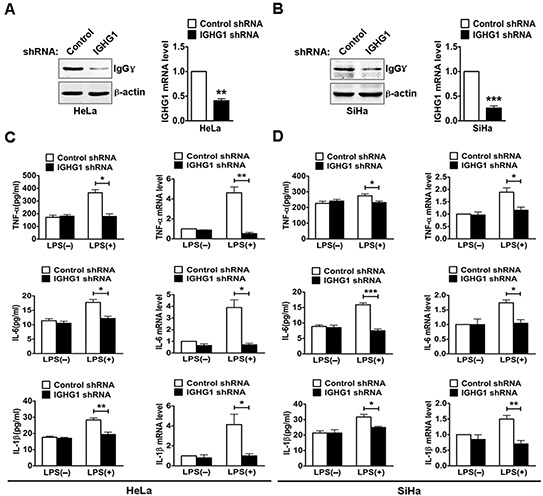
Reduction of IgG inhibited LPS induced proinflammatory cytokine production in cervical cancer cells **(A)** HeLa cells were transfected with plasmids encoding small RNAs (Control siRNA and IGHG1 siRNA), and selected under 800 μg/ml G418. The efficiency of silencing was evaluated with immunoblot (left panel) or RT-qPCR (right panel). **(B)** SiHa cells were transfected transiently with IGHG1 shRNA or control shRNA. IgGγ protein levels (left panel) and mRNA levels (right panel) were detected. HeLa cells. **(C)** and SiHa cells **(D)** treated with IGHG1 shRNA or control shRNA were stimulated with 100 ng/ml LPS for 20 h. The proinflammatory cytokines (TNF-α, IL-6, and IL-1β) were measured with ELISA (left panel) and RT-qPCR (right panel). Data are presented as means±S. D. of three independent experiments (*P<0.05; **P< 0.01; ***P<0.001).

To test whether IgG was involved in the regulation of TLR4 signaling, we established HeLa cells stably expressing immunoglobulin heavy constant gamma 1(IGHG1) shRNA or control shRNA. The efficiency of stable silencing was confirmed with immunoblot or RT-qPCR. The result showed that IgGγ expression at both protein (Fig. 2A, left panel) and mRNA (Fig. 2A, right panel) levels was significantly downregulated. We then silenced IgGγ expression in SiHa cells with transient transfection of IGHG1 shRNA and corresponding control shRNA. A significant decline of IgGγ expression at both protein (left panel) and mRNA (right panel) levels was obtained in SiHa cells (Fig. 2B). To determine the effect of IGHG1 knockdown on the production of proinflammatory cytokines including TNF-α, IL-6, and IL-1β, HeLa and SiHa cells were stimulated with or without LPS. The results indicated that the production of the proinflammatory cytokines in the stably transformed HeLa cells was significantly decreased after LPS treatment at both protein level (Fig. 2C, left panel) as examined with ELISA and mRNA level (Fig. 2C, right panel) as examined with RT-qPCR following the knockdown of IGHG1. Similar results were obtained in SiHa cells after the reduction of IGHG1 (Fig. 2D).

### Silencing of IgG inhibits LPS-initiated TLR4 signaling pathways in cervical cancer cells

**Figure 3:**
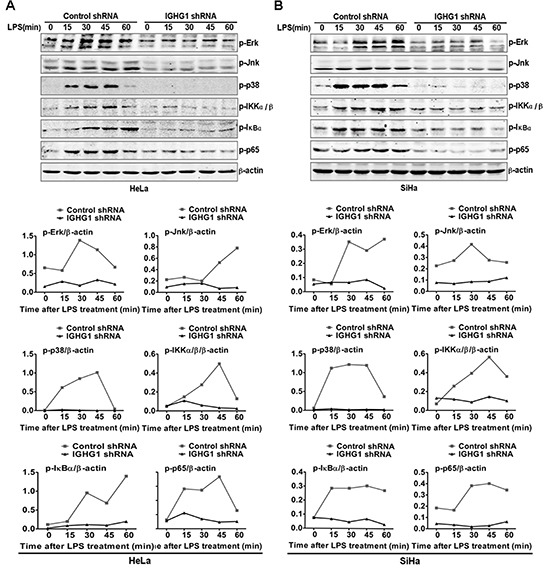
Reduction of IgG impaired LPS-initiated TLR4 signaling pathways in cervical cancer cells **(A)** Immunoblot analysis of phosphorylated (p-) signaling molecules (p-ERK, p-JNk, p-p38, p-IKKα/β, p-IκB α, and p-p65) in lysates of HeLa cells stably expressing IGHG1 shRNA or control shRNA stimulated for 0–60min (above lanes) with 100 ng/ml LPS. β-acin was derived from the same samples and used as an internal control. Similar experiments were performed using SiHa cells treated with IGHG1 shRNA or control shRNA **(B)**. The shown results are representative of three independent experiments (upper panel). Phosphorylation levels of the above proteins were quantitated by band density scanning and shown in the lower panel. The values were normalized to the β-actin signal.

To verify whether IgG was a positive regulator of TLR4 signaling, we examined the effect of IGHG1 knockdown on the activation status of MAPK (ERK, JNK, and p38) in IGHG1-silenced HeLa cells after LPS stimulation. The results showed that the activation of the above molecules was significantly decreased in IGHG1-silenced HeLa cells after LPS stimulation, compared with that of control shRNA cells (Fig. 3A). In addition, we tested the effect of IGHG1 silencing on the activation of transcription factor NF-κB pathway, which are downstream of TLR4 signaling pathways. We found that phosphorylations of inhibitory NF-κB α (IκBα), IκB kinase α/β (IKKα/β), and NF-κB subunit p65 were markedly impaired in LPS-stimulated IGHG1-deficient HeLa cells (Fig. 3A). Similar results were obtained in LPS-stimulated IGHG1- deficient SiHa cells (Fig. 3B).

### Silencing of IgG impaires LPS-induced NF-κB nuclear translocation and its responsive element activity in cervical cancer cells

**Figure 4:**
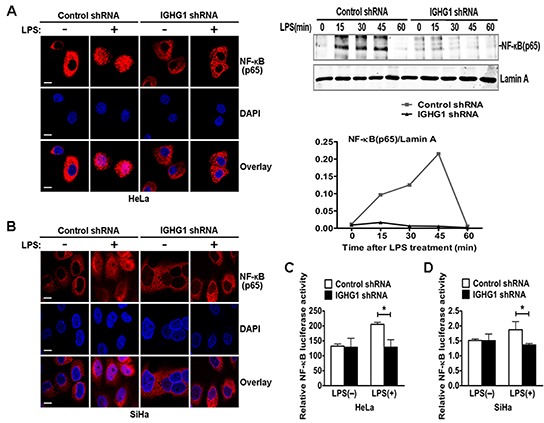
Reduction of IgG impaired LPS-Induced NF-κB activation and nuclear translocation in cervical cancer cells **(A)** HeLa cells stably expressing IGHG1 shRNA or control shRNA were treated with or without 100 ng/ml LPS for 45 minutes. The above cells were fixed and stained for NF-κB (red). Nuclei were stained with 4′, 6-diamidino-2-phenylindole (DAPI). The merged images were viewed with a confocal microscope and shown in the left panel (Scale bar, 20 μm). The above cells were treated with 100 ng/ml LPS, cell nuclear proteins were then prepared at different time points (0, 15, 30, 45, and 60 minutes) and blotted with NF-κB subunit p65 antibody. Lamin A contained in nuclear fractions was probed as quantitative controls. The results shown are representative of three independent experiments (right panel). The quantified results of the above proteins were shown in the right panel. **(C)** HeLa cells stably expressing IGHG1 shRNA or control shRNA were transiently cotransfected with pTK–Renilla-luciferase and NF-κB luciferase reporter plasmids. After 24 h of culture, the cells were treated with or without 100 ng/ml LPS for 20 h. NF-κB luciferase activity was measured using the Dual-Luciferase Reporter Assay System normalized by Renilla luciferase activity. Similar experiments were performed using SiHa cells **(B, D)**. Data are shown as mean±S.D. of three independent experiments (*P<0.05).

To further investigate whether IGHG1 deficiency inhibited the TLR4 signaling pathways in cervical cancer cells, we examined nuclear translocation of NF-κB subunit p65 in Hela cells with immunostaining after LPS treatment. The results showed that IGHG1 deficiency suppressed p65 translocation from cytoplasm to nuclear, compared with that of control shRNA HeLa cells (Fig. 4A, left panel). We also confirmed the above phenomenon by detecting the protein expression of p65 in the nucleus of HeLa cells stably expressing IGHG1 shRNA or control shRNA after LPS treatment for different time periods (Fig. 4A, right panel). We then tested the effect of IGHG1 deficiency on NF-κB luciferase activity in HeLa cells. The results indicated that IGHG1 knockdown decreased the LPS-induced transcriptional activity of NF-κB (Fig. 4C). Similar results were obtained in IGHG1 silencing SiHa cells (Fig. 4B and D).

### Recruitment of IgG to TLR4 in LPS-activated cervical cancer cells

**Figure 5:**
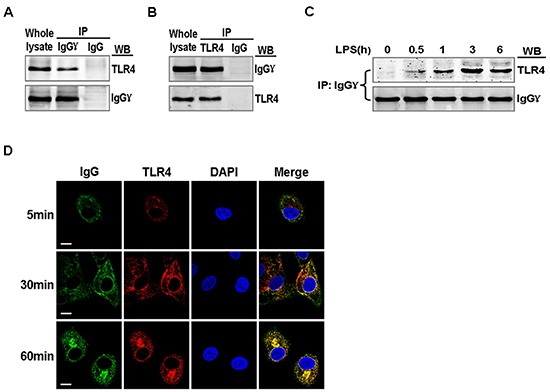
Association of TLR4 with IgG in LPS activated cervical cancer cells HeLa cells were stimulated with 100 ng/ml LPS for 1 h **(A and B)** or 0, 0.5, 1, 3, and 6 h. **(C)** Lysates of the cells were immunoprecipitated with mouse anti-human IgGγ or TLR4 or normal mouse IgG and then detected with anti-TLR4 or anti-IgGγ antibodies. The results shown are representative of three independent experiments. **(D)** HeLa cells were stimulated with 100 ng/ml LPS for 5, 30, and 60 min, then fixed and double-stained for IgGγ (green) and TLR4 (red). Nuclei were stained with DAPI. The merged images (yellow) were viewed with a confocal microscope and shown (Scale bar, 20 μm).

We explored the mechanism by which IgG positively regulated LPS-induced proinflammatory cytokine production in cervical cancer cells. We investigated whether TLR4 could associate with IgG in cervical cancer cells. After being activated by LPS, TLR4 interacted with IgG (Fig. 5A and B). Further studies showed that the coprecipitation between TLR4 and IgG was enhanced after LPS stimulation and reached the peak level at 3 h, then gradually decreased (Fig. 5C). Confocal immunofluorescence microscopy revealed that TLR4 colocalized with IgG in the cytoplasm of HeLa cells, and the colocalization was significantly enhanced with the treatment of LPS (Fig. 5D).

### Silencing of IgG downregulates TLR4 expression

**Figure 6:**
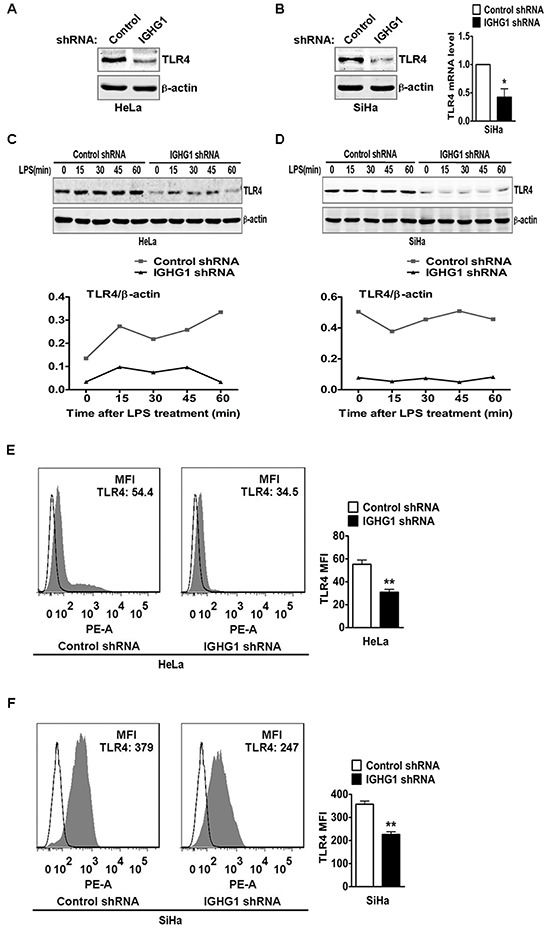
Reduction of IgG downregulated TLR4 expression TLR4 expression at protein level in HeLa cells stably expressing IGHG1 shRNA or control shRNA. **(A)** and IGHG1 silenced SiHa cells or control cells. **(B)** were measured with immunoblot using anti-TLR4 Antibody. β-actin was used as a quantitative control. TLR4 expression at mRNA level in the above SiHa cells was also measured with RT-qPCR **(B)**. **(C)** HeLa cells stably expressing IGHG1 shRNA or control shRNA and IGHG1 silenced SiHa cells or control cells **(D)** were treated with 100 ng/ml LPS. Dynamic changes of TLR4 expression (0, 15, 30, 45, and 60 minutes) were examined with immunoblot using TLR4 antibody. β-actin was used as quantitative control. The results shown are representative of three independent experiments (upper panel). The quantified results of TLR4 expression were shown in the lower panel. The values were normalized to the β-actin signal. TLR4 expressions on plasma membrane in HeLa cells stably expressing IGHG1 shRNA or control shRNA **(E)** and IGHG1-silenced or control vector-silenced SiHa cells **(F)** were detected with phycoerythrin-conjugated TLR4 antibody by flow cytometry. The mean fluorescence intensity (MFI) was calculated and shown at the right panel. The data are expressed as mean±S.D. from three independent experiments (*P<0.05; **P<0.01).

Numerous studies have shown that regulation of TLR4 signaling is mostly dependent on the regulation of TLR4 expression levels [22-28]. Therefore we postulated whether IgG positively regulated LPS-induced proinflammatory cytokine production, possibly through its effects on TLR4 expression. We examined TLR4 expression in cervical cancer cells after IGHG1 silencing. We found that IGHG1 silencing significantly downregulated TLR4 expression at protein level in HeLa cells (Fig. 6A) and SiHa cells (Fig. 6B, left panel). In addition, IGHG1 silencing did not affect TLR4 expression at mRNA level in HeLa cells (data not shown) but reduced that in SiHa cells (Fig. 6B, right panel). To further test the effects of IgG on TLR4 expression, we observed TLR4 dynamics after LPS treatments of cervical cancer cells. The results showed that the overall TLR4 expression was at a high level from 0 to 60 minutes in control shRNA Hela cells, whereas that in IGHG1-silenced Hela cells remained at a low level (Fig. 6C). Similar results were obtained in IGHG1-silenced SiHa cells and their control cells (Fig. 6D). Flow cytometric analysis demonstrated that the TLR4 expression level in the plasma was also decreased in IGHG1-dowregulated Hela (Fig. 6E) and SiHa cells (Fig. 6F).

### TLR4 regulates LPS-induced proinflammatory cytokine production of IgG-deficient cervical cancer cells

**Figure 7:**
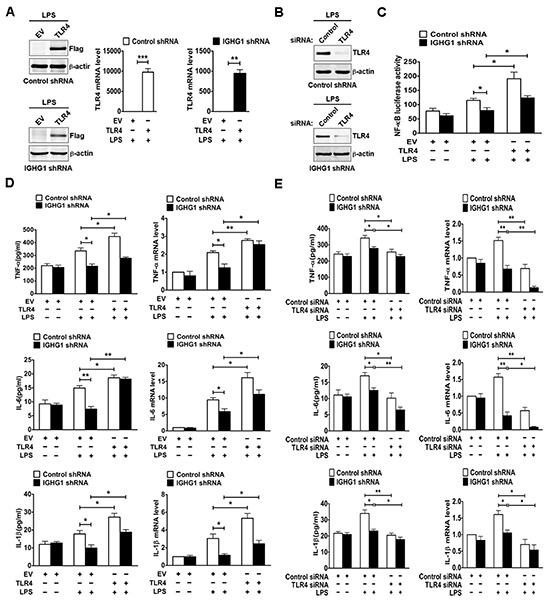
Reduction of IgG inhibited LPS-induced proinflammatory cytokine production through downregulating TLR4 expression in cervical cancer cells **(A)** HeLa cells stably expressing IGHG1 shRNA or control shRNA were transiently transfected with empty vector or Flag-tagged TLR4 plasmids respectively. Overexpression of TLR4 was measured with immunoblot (left panel) or RT-qPCR (right panel). **(B)** The above stably transformed cells were transfected transiently with TLR4 siRNA or control siRNA. TLR4 protein levels were detected. **(C)** The above stably transformed cells concomitantly overexpressing TLR4 were transiently cotransfected with pTK–Renilla-luciferase and NF-κB luciferase reporter plasmids. After 24 h of culture, the cells were treated with or without 100 ng/ml LPS for 20 h. NF-κB luciferase activity was measured using the Dual-Luciferase Reporter Assay System normalized against Renilla luciferase activity. The above stably transformed cells concomitantly overexpressing TLR4 **(D)** or downregulating TLR4 expression **(E)** were treated with or without 100 ng/ml LPS for 20 h. The proinflammatory cytokines (TNF-α, IL-6, and IL-1β) were measured with ELISA (left panel) and RT-qPCR (right panel). Data are presented as means±S. D. of three independent experiments (*P<0.05; **P< 0.01; ***P< 0.001).

We further evaluated the role of TLR4 in IgG enhanced LPS-induced proinflammatory cytokine production of cervical cancer cells. After testing TLR4 expression at both protein and mRNA levels with immunoblot (Fig. 7A left panel) and RT-qPCR (Fig. 7A right panel), we measured NF-κB luciferase activity and the production of proinflammatory cytokines including TNF-α, IL-6, and IL-1β. The results showed that IGHG1 knockdown decreased LPS-induced transcriptional activity of NF-κB (Fig. 7C) and the production of the above proinflammatory cytokines at both protein and mRNA levels (Fig. 7D), whereas overexpression of TLR4 completely reversed the above effects by recovering LPS-induced proinflammatory cytokine production. In addition, we examined the production of the above proinflammatory cytokines at both protein and mRNA levels after TLR4 downregulation by siRNA silencing in the above stably transformed cells. Our results indicated that TLR4 downregulation enhanced the reduction of the above proinflammatory cytokine production, which was induced by IGHG1 knockdown, through further inhibiting LPS-induced signaling (Fig. 7B and E).

### Silencing of IgG reduces the production of LPS-induced proinflammatory cytokines *in vivo*

**Figure 8:**
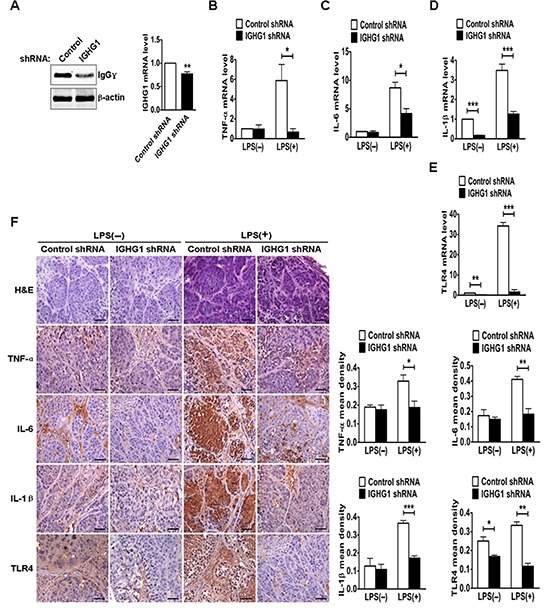
Reduction of IgG reduced LPS-induced proinflammatory cytokine production *in vivo* **(A)** The protein and mRNA of IgGγ in tumor tissues from the above different groups of mice, including group 1 (injection of control shRNA cells), group 2 (injection of control shRNA cells with LPS treatment), group 3 (injection of IGHG1 shRNA cells) and group 4 (injection of IGHG1 shRNA cells with LPS treatment), were measured with immunoblot or RT-qPCR. **(B-E)** The mRNA of proinflammatory cytokines (TNF-α, IL-6, and IL-1β) and TLR4 in tumor tissues from the above different groups of mice were measured with RT-qPCR. **(F)** H&E, TNF-α, IL-6, IL-1β, and TLR4 staining (scale bar, 20 μm) of tumor tissues from the above different groups of mice. The mean densities of above protein molecules were analyzed and shown in the right panel. The RT-qPCR and mean density data are expressed as mean±S.D. from three independent experiments (*P<0.05; **P<0.01; ***P<0.001).

To test the *in vivo* effects of IgG on LPS-induced proinflammatory cytokine production, we established a mouse model of endotoxemia. We first examined IgG expression of different tumor tissues. The results showed that IgGγ expressions of tumor tissues from group 3 (IGHG1 shRNA) or 4 (IGHG1 shRNA with LPS treatment) were lower than that from group 1 (Control shRNA) or 2 (Control shRNA with LPS treatment) at both protein and mRNA levels (Fig. 8A). RT-qPCR analysis indicated that the expressions of the proinflammatory cytokines (TNF-α, IL-6, and IL-1β) and TLR4 of tumor tissues from group 2 were markedly increased at mRNA level after LPS treatment, compared with those of group 1 or 3 without LPS treatment. In addition, the expressions of these proinflammatory cytokines and TLR4 of tumor tissues from group 4 were significantly decreased after LPS treatment, compared with those from group 2 (Fig. 8B-E). Similar results at protein level were obtained with immunohistochemistry (Fig. 8F).

### IgG and TLR4 expressions in human cervical cancer are closely related with proinflammatory cytokines

**Figure 9:**
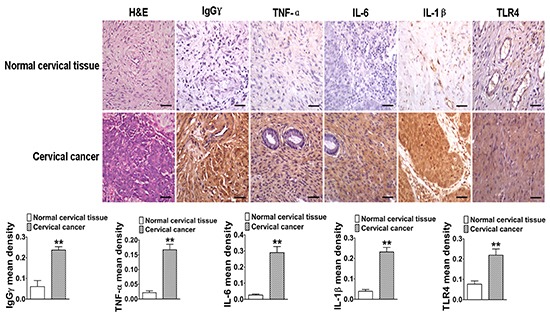
The close relationship between IgG, TLR4, and proinflammatory cytokines in human cervical cancer The analytic comparison of IgG, TNF-α, IL-6, IL-1β, and TLR4 expressions (scale bar, 20 μm) in cervical cancer tissues and normal cervical tissues. Their mean densities were calculated and presented in the lower panel. Data shown are the mean±S.D. of three independent experiments (*P<0.05; **P<0.01).

To confirm that TLR4 expression played a key role in IgG positively regulated LPS-induced proinflammatory cytokine production, we analyzed TLR4 expression in human cervical cancer tissues and normal cervical tissues with immunohistochemistry. The results showed that TLR4 mean density of cervical cancer tissues was higher than that of normal cervical tissues (Fig. 9). After analysis of H&E staining, the expressions of IgGγ and proinflammatory cytokines (TNF-α, IL-6, and IL-1β) were also examined. Immunohistochemistry results indicated that the expressions of the above proteins were upregulated in cervical cancer tissues, compared with normal cervical tissues (Fig. 9).

## DISCUSSION

In this study, we demonstrated that the expression of cancer-derived IgG increased after exposure to LPS initially and then decreased afterwards in cervical cancer cells, suggesting that IgG expression was regulated by LPS stimulation in cervical cancer cells. Reduction of IgG attenuated LPS-induced proinflammatory cytokine production in cervical cancer cells. The phosphorylation levels of NF-κB and MAPK (ERK, JNK, and p38) were consistently suppressed by knockdown of IgG in cervical cancer cells, which in turn impaired NF-κB nuclear translocation and the activity of NF-κB responsive element. Furthermore, we found that IgG was recruited to TLR4 in the cytoplasm after LPS stimulation, and reduction of IgG inhibited LPS-initiated signaling pathways through downregulating TLR4 expression. Similar results were obtained *in vivo*. Briefly, these findings demonstrate that cancer-derived IgG is a positive regulator of LPS-induced proinflammatory cytokine production via binding to TLR4 in cervical cancer cells.

Accumulated evidence suggests that TLR4 is expressed not only by immune cells but also by a variety of cancer cells such as cervical cancer, breast cancer, and lung cancer [29-33]. TLR4 is a double-edged sword in carcinogenesis. On the one hand, TLR4, as a negative regulator of cancer, inhibits tumor growth and progression. TLR4 expressed in cancer can prevent infection by microbial pathogens such as hepatitis B and C viruses [34, 35], Helicobacter pylori [36, 37], and human papilloma virus [38], which may cause cancer. High dose of LPS, TLR4 ligand, can directly kill tumor cells so that it has been used to treat colorectal and lung cancer in Phase II clinical trials [39]. TLR4 activation may also lead to tumour regression by increasing vascular permeability [40]. On the other hand, TLR4 stimulation promotes tumorigensis. TLR4 can upregulate the NF-κB signaling and produce anti-apoptotic proteins that promote carcinogenesis and cancer cell growth and proliferation [30, 41, 42]. When the function of TLR4 signaling pathways is gradually unveiled and confirmed in cancer cells, their regulation attracts much attention. In the past few decades, many regulators of TLR4 signaling pathways, including both positive and negative ones, have been found [43-46]. It was reported that Rab10 positively regulates TLR4 signaling pathways by enhancing replenishment of TLR4 onto the plasma membrane. On the contrary, Rab7b negatively regulates TLR4 signaling pathways in macrophages through promoting lysosomal degradation of TLR4, which leads to decrease of TLR4 expression [47]. In our study, LPS regulated kinetically IgG expression in cervical cancer cells. Reduction of IgG inhibited the production of LPS-induced proinflammatory cytokines (TNF-α, IL-6, and IL-1β). It should be pointed that we selected IGHG1 for the silencing target only rather than IGHG2, IGHG3 and so on because knockdown of endogenous IGHG1 could lead to more significant biological effects than that of others in cervical cancer cells according to our preliminary experiments. In brief, the above results indicated that IgG was involved in positive regulation of TLR4 signaling in cervical cancer cells.

TLR4 is one of the key pattern-recognition receptors which recognize the conserved components of pathogens in the innate immune system, and it has a critical role in host defense against deleterious microbial infection [48, 49]. After LPS stimulation, TLR4 initiates innate immune responses by activating signaling pathways including MyD88-dependent and -independent (or TRIF dependent) pathways and consequently induces the production of proinflammatory cytokines and type I interferon [50]. Our results demonstrated that cancer-derived IgG silencing inhibited the phosphorylation levels of MAPK (ERK, JNK, and p38), IKKα/β, IκBα, and NF-κB subunit p65 in cervical cancer cells. In addition, knockdown of IgG also reduced both the luciferase activity of NF-κB and the production of proinflammatory cytokines (TNF-α, IL-6, and IL-1β). On the contrary, the activation of IRF3 and the expression of its downstream target IFNβ were not impaired by IgG silencing (data not shown). Taken together, the above results indicated that IgG had different regulatory mechanism on TLR4 signaling pathways in cervical cancer cells.

TLR4, as a starting point, plays a vital role in the entire LPS initiated signaling pathways. It was reported that reduction of TLR4 expression led to hyposensitivity of macrophages to LPS signaling, which eventually impaired the production of proinflammatory cytokines [47]. After downregulation of TLR4 expression by TLR4 short interfering RNA or blockade TLR4 with a cell permeable TLR4 inhibitory peptide, the effects induced by LPS such as the production of nitric oxide, IL6, and IL12 were almost abolished [16]. Our results demonstrated that IgG knockdown reduced the production of LPS-induced proinflammatory cytokines in cervical cancer cells. In the meantime, IgG knockdown also downregulated TLR4 expression in cervical cancer cells. To verify whether the promoting effects of IgG on LPS-induced proinflammatory cytokine production were due to the increase of TLR4 expression in cervical cancer cells, we upregulated or downregulated TLR4 expression in HeLa cells stably expressing IGHG1 shRNA or control shRNA and activated the signaling pathway by LPS stimulation. The results showed that the reduction of LPS-induced proinflammatory cytokines production in IGHG1 shRNA cells was reversed significantly due to the overexpression of TLR4. In addition, the production of the above proinflammatory cytokines in IGHG1 shRNA cells was also further downregulated after the reduction of TLR4 expression. Similar results were obtained in animal model and human cervical cancer tissues. Our results were in support of the above reports. Previous studies demonstrated that cervical cancer tissues expressed TLR4 and proinflammatory cytokines at high level, compared to normal cervical tissues [51-57]. Similar results were obtained in our *in vivo* experiments. TLR4 signaling pathways consist of many important components other than TLR4. They include MyD88, toll-il-1 receptor domain-containing adapter protein (TIRAP), TRIF, toll-like receptor adaptor molecule 2 (TRAM), interleukin-1 receptor-associated kinase 1 (IRAK1), TNF receptor-associated factor 6 (TRAF6) and so on. It is possible that each of these components can be the target of regulation of TLR4 signaling pathways. Western blot results showed that IgG did not affect the protein levels of MyD88, IRAK1, and TRAF6 (data not shown), indicating that TLR4 was at least the major target involved in IgG-mediated positive regulation of LPS-induced proinflammatory cytokine production in cervical cancer cells. Indeed, reports about the regulation of TLR4 or other TLRs signaling pathways in cancer cells have been few.

Our previous study demonstrated that HeLa cells stably expressing IgG grew faster than IGHG1 shRNA-treated cells *in vitro* and *in vivo*, indicating that cancer-derived IgG can promote tumor cell growth and proliferation [21]. Our present results show that IgG is recruited to TLR4 and elevates its expression to boost TLR4 signaling which was reported to enhance cervical cancer cell proliferation and anti-apoptosis [57, 58]. The above results strongly suggest that IgG may promote cervical cancer cell growth and proliferation through enhancing LPS-induced proinflammatory cytokine production via binding to TLR4. In other words, the physiological significance of IgG in cancer immunology is that it may contribute to the development of cervical cancer through positively regulating TLR4 signaling pathways. It should be pointed out that cancer-derived IgG is produced in both cytoplasmic and secreted forms [14]. In this study, we only focused on the cytoplasmic form of IgG. In summary, cancer-derived IgG promotes LPS-induced proinflammatory cytokine production via interacting with TLR4 and enhancing its expression. The proposed mechanism provides a new clue to the possible functions of cancer-derived IgG, which may also serve as a novel therapeutic target in treating inflammation-mediated cancer. On the one hand, we may design and synthesize effective lentivirus-mediated IGHG1 shRNA according to IGHG1 gene sequence. These lentivirus particles reduce IgG expression by directly injecting into the tumor body and infecting tumor cells, which will lead to down-regulation of TLR4 expression according to our results. Finally, carcinogenesis will be prevented or slowed down due to the suppression of inflammation. On the other hand, we may screen specific anticancer agents such as chemicals or cytokines, which suppress TLR4 expression to reduce inflammation by specifically inhibiting IgG expression, to kill cancer cells by directly injecting them into the tumor body.

## MATERIALS AND METHODS

### Mice and reagents

BALB/c mice (4-6 weeks old) were purchased from Vital River Laboratories (Beijing, China). All animal experiments were performed in accordance with the guidelines of Shantou University Medical College. LPS (from *Escherichia coli* 0111:B4) was purchased from Sigma (St Louis, MO, USA) and repurified as described previously [22, 23]. The enzyme-linked immunoassay kits for TNF-α, IL-6, and IL-1β were from Meilian Biotechnology (Shanghai, China). Flag-tagged TLR4 plasmid and NF-κB luciferase plasmid were gifts from Dr Y. Eugene Chin (Brown University School of Medicine-Rhode Island Hospital, USA). The pRL-TK–Renilla-luciferase plasmid was a gift of Dr Xiangwei Gao (Zhejiang University School of Medicine, China). The primary and second antibodies were shown in Supplementary Table 1.

### Cell culture, human tissue specimens and transfection

The human cervical cancer cell lines HeLa and SiHa were obtained from the ATCC. The above cell lines were cultured in Dulbecco's modified Eagle's medium (Invitrogen, Carlsbad, CA, USA) with 10% fetal bovine serum (Hyclone/Thermo Fisher Scientific Inc., Waltham, MA, USA) at 37°C in a humidified atmosphere with 5% CO_2_. The stably transformed HeLa cells were selected with 800 mg/ml of antibiotic G-418 (BBI, Toronto, ON, Canada). Human cervical squamous cell carcinoma tissues (International Federation of Gynecology and Obstetrics (FIGO) stage IIIB) and adjacent normal tissues were obtained from Cancer Hospital of Shantou University Medical College. The study was approved by the Ethical Committee of Shantou University Medical College. Transfections using X-tremeGENE HP DNA Transfection Reagent (Roche, Basel, Switzerland) were performed according to manufacturer's instructions.

### Immunoprecipitation, western blot and extraction of nuclear proteins

Immunoprecipitation and western blot were performed as previously described [21]. Nuclear proteins were extracted by NE-PER Nuclear and Cytoplasmic Extraction Reagents (Pierce, Rockford, IL, USA) according to the manufacturer's instructions. Cell extracts were subjected to western blot analysis. The integrated optical density of IgGγ, NF-κB subunit p65, TLR4, and phosphorylations of ERK, JNK, p38, IKKα/β, IκBα, and NF-κB subunit p65 were quantified using Gel-Pro analyzer software.

### Immunohistochemistry and immunofluorescence

Immunohistochemistry was performed as previously described [21]. The mean densities of IgGγ, TNF-α, IL-6, IL-1β, and TLR4 were quantified using Image-Pro Plus software. Immunofluorescence was performed as previously described [15]. Cells were imaged using an Olympus confocal imaging system (Olympus, Tokyo, Japan).

### RNA purification and RT-qPCR

Total RNA was isolated using Trizol reagent (Takara, Tokyo, Japan) following the manufacturer's protocol. Reverse transcription of total RNA was performed using PrimeScript RT reagent Kit (Perfect Real Time) (Takara). RT-qPCR analysis was performed using the ABI7500 (Applied Biosystems, Foster City, CA, USA). The primers were shown in Supplementary Table 2.

### RNA interference

siRNAs were transfected into human cervical cancer cell lines HeLa and SiHa with X-tremeGENE siRNA Transfection Reagent (Roche Applied Science, Indianapolis, IN, USA). Cells were further analyzed 72 h after transfection. IGHG1 and TLR4 siRNA sequence was as described previously [21, 59].

### Measurement of luciferase reporter activity and proinflammatory cytokines

Luciferase reporter assay was performed as previously described [47]. TNF-α, IL-6, and IL-1β in cell supernatants were measured with ELISA kits (Meilian Biotechnology, Shanghai, China) according to the manufacturer's instructions.

### Flow cytometric analysis

Flow cytometric analysis was performed as previously described [47]. Data were processed with Flowjo 7.6 software (Treestar, Ashland, OR, USA).

### Establishment of a mouse model of endotoxemia

24 BALB/c nude mice were randomly divided into 4 groups, each had 6 mice. Group 1 or 2 were injected subcutaneously with control shRNA cells (1×10^7^ in a volume of 100 μl normal saline) into the bilateral groin, group 3 or 4 were injected subcutaneously with IGHG1 shRNA cells (1×10^7^ in a volume of 100 μl normal saline) into the bilateral groin. Tumor growth was monitored externally using vernier calipers for 8 weeks. After 8 weeks, LPS (10 mg/ kg body weight) was directly injected into the tumor body (group 2 and 4) as experimental groups, while normal saline was injected into the tumor body (group 1 and 3) as control groups. After 6 h, all mice were killed, their tumors were removed with one part fixed in 10% formalin and another stored at −70°C.

### Statistical analysis

SPSS software (version 16.0; SPSS, Inc., Chicago, IL, USA) was used for statistical analysis. All experiments were repeated at least three times. The data were expressed as mean±S.D. and compared with independent-samples *t*-test. Statistical significance was determined as P<0.05.

## SUPPLEMENTARY TABLES



## Abbreviations

IgG: immunoglobulin
TLR4: toll-like receptor 4
MyD88: myeloid differentiating factor 88
TRIF: toll/il-1r domain-containing adaptor inducing interferon-β
NF-κB: nuclear factor κB
MAPK: mitogen-activated protein kinase
TNF-α: tumor necrosis factor
IL: interleukin
IRF3: interferon regulatory factor 3
IFN: interferon
V_H_DJ_H_: the heavy chain variable segment (V_H_), diversity segment (D), and joining segment (J_H_) recombination
Oct-2: octamer-related protein-2
NeuAc: N-acetylneuraminic acid
LPS: lipopolysaccharide
ERK: extracellular signal-regulated kinase
JNK: c-Jun N-terminal kinase
RT-qPCR: real-time quantitative reverse transcription PCR
IGHG1: immunoglobulin heavy constant gamma 1
IKKα/β: IκB kinase α/β
IκBα: inhibitory NF-κB α
IRAK1: interleukin-1 receptor-associated kinase 1
TRAF6: TNF receptor-associated factor 6
DAPI: 4′, 6-diamidino-2-phenylindole
