# Supplementary material for: Cancer-derived immunoglobulin G promotes LPS-induced proinflammatory cytokine production via binding to TLR4 in cervical cancer cells
Source: Oncotarget. 2014 Aug 23;5(20):9727–43. doi: 10.18632/oncotarget.2359 (PMC4259433; doi:10.18632/oncotarget.2359)
Supplement: Supplementary file 1 [file oncotarget-05-9727-s001.pdf]

Supplementary Table S1. Details of Primary and Second Antibodies.

Primary antibody	Source	Dilution	Incubation	Supplier
IgG (γ chain specific)	Rabbit	1:500	4°C, overnight	Dako, Denmark
phosphothreonine ERK-1/2	Mouse	1:500	4°C, overnight	Sigma-Aldrich, USA
Phospho-SAPK/JNK (Thr183/Tyr185)	Mouse	1:500	4°C, overnight	Cell Signaling Technology, USA
Phospho-p38 MAPK (Thr180/Tyr182)	Rabbit	1:500	4°C, overnight	Cell Signaling Technology, USA
Phospho-Ikkα/β (Ser176/180)	Rabbit	1:500	4°C, overnight	Cell Signaling Technology, USA
Phospho-IκBα (Ser32)	Rabbit	1:500	4°C, overnight	Cell Signaling Technology, USA
Phospho-NF-κB p65 (Ser536)	Rabbit	1:500	4°C, overnight	Cell Signaling Technology, USA
NF-κB p65	Rabbit	1:500	4°C, overnight	Cell Signaling Technology, USA
TLR4	Mouse	1:500	4°C, overnight	Abcam, USA
TLR4 (PE)	Mouse		4°C, 30min	Abcam, USA
Flag	Rabbit	1:1000	4°C, overnight	GenScript, USA
TNF-α	Rabbit	1:100	4°C, overnight	BOSTER, China
IL-6	Rabbit	1:100	4°C, overnight	BOSTER, China
IL-1β	Rabbit	1:100	4°C, overnight	BOSTER, China
β-actin	Rabbit	1:500	4°C, overnight	Sangon Biotech, China
Lamin A	Rabbit	1:500	4°C, overnight	BOSTER, China
**Second antibody**				
Alexa Fluor® 594-Conjugated AffiniPure Goat Anti-Rabbit IgG (H+L)		1:300	Room temperature, 1h	ZSGB-BIO, China
Alexa Fluor® 488-Conjugated AffiniPure Goat Anti-Rabbit IgG (H+L)		1:300	Room temperature, 1h	ZSGB-BIO, China
IRDye 800 CW goat anti-mouse		1:15000	Room temperature, 1h	LI-COR Biosciences, USA
IRDye 680 CW goat anti-mouse		1:15000	Room temperature, 1h	LI-COR Biosciences, USA
IRDye 800 CW goat anti-rabbit		1:15000	Room temperature, 1h	LI-COR Biosciences, USA
IRDye 680 CW goat anti-rabbit		1:15000	Room temperature, 1h	LI-COR Biosciences, USA

Supplementary Table S2. RT-qPCR Primers

Gene name	RT-qPCR primers	Primer sequence 5′ -3′	Size (bp)
IGHG1	Sense	ACTCCGACGGCTCCTTCTTC	20
	Antisense	TTCTGCGTGTAGTGG TTGTGC	21
TNF-α	Sense	CCTCTCTCTAATCAGCCCTCTG	22
	Antisense	GAGGACCTGGGAGTAGATGAG	21
IL-6	Sense	ACTCACCTCTTCAGAACGAATTG	23
	Antisense	CCATCTTTGGAAGGTTCAGGTTG	23
IL-1β	Sense	ATGATGGCTTATTACAGTGGCAA	23
	Antisense	GTCGGAGATTCGTAGCTGGA	20
TLR4	Sense	AGTTGATCTACCAAGCCTTGAGT	23
	Antisense	GCTGGTTGTCCCAAAATCACTTT	23
β-actin	Sense	AGCGAGCATCCCCCAAAGTT	20
	Antisense	GGGCACGAAGGCTCATCATT	20
